# A Landscape and Climate Data Logistic Model of Tsetse Distribution in Kenya

**DOI:** 10.1371/journal.pone.0011809

**Published:** 2010-07-27

**Authors:** Nathan Moore, Joseph Messina

**Affiliations:** 1 Department of Environmental and Resource Sciences, Zhejiang University, Hangzhou, China; 2 Department of Geography, Center for Global Change and Earth Observations, Michigan State University, East Lansing, Michigan, United States of America; Stanford University, United States of America

## Abstract

**Background:**

*Trypanosoma spp*, biologically transmitted by the tsetse fly in Africa, are a major cause of illness resulting in both high morbidity and mortality among humans, cattle, wild ungulates, and other species. However, tsetse fly distributions change rapidly due to environmental changes, and fine-scale distribution maps are few. Due to data scarcity, most presence/absence estimates in Kenya prior to 2000 are a combination of local reports, entomological knowledge, and topographic information. The availability of tsetse fly abundance data are limited, or at least have not been collected into aggregate, publicly available national datasets. Despite this limitation, other avenues exist for estimating tsetse distributions including remotely sensed data, climate information, and statistical tools.

**Methodology/Principal Findings:**

Here we present a logistic regression model of tsetse abundance. The goal of this model is to estimate the distribution of tsetse fly in Kenya in the year 2000, and to provide a method by which to anticipate their future distribution. Multiple predictor variables were tested for significance and for predictive power; ultimately, a parsimonious subset of variables was identified and used to construct the regression model with the 1973 tsetse map. These data were validated against year 2000 Food and Agriculture Organization (FAO) estimates. Mapcurves Goodness-Of-Fit scores were used to evaluate the modeled fly distribution against FAO estimates and against 1973 presence/absence data, each driven by appropriate climate data.

**Conclusions/Significance:**

Logistic regression can be effectively used to produce a model that projects fly abundance under elevated greenhouse gas scenarios. This model identifies potential areas for tsetse abandonment and expansion.

## Introduction

### Tsetse background

Trypanosomiasis is a neglected tropical disease that currently represents a major threat to African cattle, costing ∼US$1.3 billion per year [1]. The vector for trypanosomiasis, the tsetse fly (*Glossina spp*), requires a habitat strongly influenced by ecological and climatic features [2], [3] — and particularly soil moisture — all of which are influenced by rainfall, soil type, temperature, and other climate variables. Tsetse flies can be categorized into three major subgenera: *palpalis* (riverine), *fusca* (forest), and *morsitans* (savanna). The most common variety of tsetse in East Africa, the *morsitans* group, primarily feeds on wildlife and cattle, and only occasionally on humans. Infection with trypanosomes may result in clinical disease, known as sleeping sickness in humans and nagana in cattle. However, infection in cattle often goes undiagnosed, and small-area studies show difficulty in relating incidence in cattle with tsetse challenge [4].

**Figure 1:**
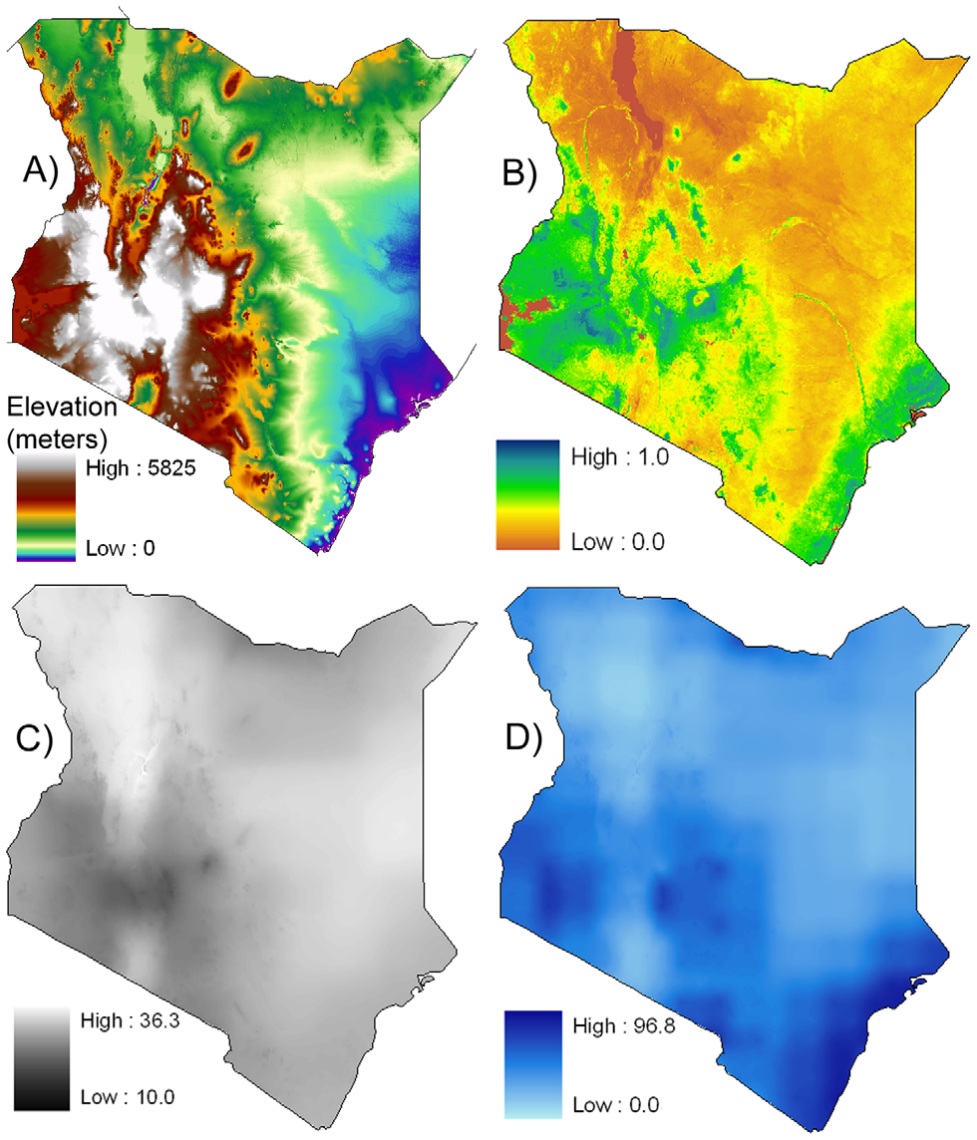
Driving variables. (A) elevation, (B) renormalized NDVI, (C) 2000 maximum temperature, (D) 2000 annual precipitation.

Kenya has a long history of tsetse infestation, and with it a heavy economic toll. Livestock production accounts for roughly 8% of Kenya's GDP. In endemic areas, tsetse control could increase livestock productivity by as much as 52% [1], [5]. Historical evidence indicates that various attempts to control or eradicate the flies have been hampered by a variety of socioeconomic and geographic factors. Among these geographic factors is elevation (Fig. 1 (A)), which is associated with numerous habitats and microclimate zones at different elevations. These microclimates, often associated with scattered tree cover, offer tsetse flies seasonal refugia and access to migrating host species. Specific tree species have long been connected to tsetse-infested areas [6], and recent work has shown that specific vegetation structures and geometry are sought by tsetse flies [7]; woody savanna land cover is especially favored [8]. Such habitats can be identified in part through the remotely sensed Normalized Difference Vegetation Index (NDVI) (Fig. 1 (B)) and maximum temperature (Fig. 1(C). Fly larvae can die as a result of drying soils. Temperature extremes, particularly above ∼36°C and below ∼10°C, also lead to adult fly mortality through starvation and water loss via respiration [9], [10]. Low humidity — moisture levels directly related to precipitation (Fig. 1 (D) — is also involved in fly mortality, though the exact mechanism is not clear [9], [11], [12]. Other important factors affecting tsetse abundance include savanna canopy cover (where flies retreat during daytime heat), presence/absence of host species (notably cattle and/or wildlife [13]–[15]), and distance to seasonal refugia. McDermott et al. [16] optimistically noted that increasing human populations could lead to slight declines in tsetse population through habitat loss, particularly in the Lake Victoria basin region. Despite these numerous factors influencing tsetse presence, estimating fly distributions has been difficult.

### Overview of previous efforts and methods

Several efforts to express the relationships between tsetse abundance, climate and socioeconomic variables have been attempted, with mixed results. A Kenya-wide 1973 survey of tsetse distribution by species based on field reports and other sampling [2] is considered among the best maps of fly distribution (hereafter, “1973 data”), but that map is now over 30 years old. More commonly, modeling approaches have been used to estimate fly populations, often using remotely sensed data [17], [18], [19]. Several simple linear models [20], [21] have been developed, largely with annually averaged data, to identify fly habitats and maximum fly belt extents. More recent research by the Food and Agriculture Organization of the United Nations (FAO) together with the International Atomic Energy Agency (IAEA) (hereafter, “2000 FAO data”) [3], [22], [23] employed logistic regressions of remotely sensed data for predicting tsetse abundance to produce a “snapshot” of tsetse distribution at a given point in time. While all of these methods have tried to connect annual data to tsetse distribution, they are hampered by a variety of factors, including irreproducible methods, statistical approaches hampered by excessive multicollinearity, and poor availability or absence of observed data — particularly with respect to tsetse and animal populations. Human population growth also affects land use, primarily through agricultural expansion, which leads to declining tsetse habitat (excluding *palpalis* species) [24]. Significant shifts in tsetse distribution by 2040 are predicted throughout Africa with a ∼7% decrease in overall tsetse population [24]. Another hampering factor is seasonal climate; since weather is highly variable in Kenya, tsetse distributions vary similarly. Despite these limitations, almost all of these studies have focused on identifying areas suitable for tsetse survival, and thus implicitly focus on regions where the average annual climate is suitable for tsetse flies to live. However, both climate and climate variability are showing signs of change due to regional climate shifts [16]. If these recent patterns of climate change are responsible for shifts in tsetse distribution, projections of climate change can then be employed to identify areas at particular risk for increased tsetse abundance.

These potential shifts in climate regimes motivate this study. Kenya's government agencies need specific projections of tsetse habitat to establish monitoring and control activities. Given that no published logistic regression model has been made available, and given the need for high-resolution projections, we present here a new model complete with maps of potential tsetse expansion.

### Purpose of the study

The objective of this study was to estimate the distribution of tsetse flies using a regression model based on remotely sensed data and climate data. No similar model of tsetse abundance as a function of climate data has been presented in the peer-reviewed literature. Our second and parallel objective is to develop a logistic regression model (LM) to incorporate measures of climate variables for predicting future tsetse-susceptible trends in distribution. Ultimately, the final product of this study was to produce a map of regions that may be newly susceptible to tsetse expansion under anticipated variable climate conditions due to greenhouse gases (GHG).

## Results

### Model construction with 1973 data

Several different independent variables were used to construct the model; a correlation matrix and basic statistics concerning these parameters' roles in the model are given in Table 1 and Table 2 respectively. This set of variables had the best AIC (Akaike Indicator Criterion) score. Most of the variables are significant based on the p-value with the exception of NDVI for May. ***Bold italics*** in Table 2 are where the standard error is more than 25% the value of the estimated coefficient, which indicates that those variables are often not predictive of tsetse abundance. Land cover (a binary suitable/unsuitable map) was a strong predictor; it also had a relatively large standard error. Other variables and their interaction terms may in some ways replace the role elevation and other omitted terms might have played in predicting tsetse abundance. Wet days and precipitation, which at first glance should be exceedingly collinear, were selected to represent separately the driest months (in the cases of September wet days and February wet days) and rainy periods (in the cases of March+April+May (MAM) precipitation—the “long rains”— and October+November+December (OND) precipitation—the “short rains”. We stress that this is a predictive, not explanatory model, and under such circumstances this is acceptable.

**Table 1:** Correlation Matrix for the broad set of variables considered.

ELEV	−0.01	0.37	0.50	0.53	0.53	0.19	0.39	0.45	0.50	0.39	−0.78	−0.68
−0.01	LC	−0.04	0.01	0.03	0.05	−0.05	0.01	−0.05	0.00	−0.03	0.03	0.03
0.37	−0.04	COW	0.39	0.37	0.36	0.26	0.29	0.17	0.35	0.29	−0.39	−0.33
**0.50**	0.01	0.39	ND2	0.91	0.87	0.56	0.30	0.24	0.42	0.49	−0.57	−0.52
**0.53**	0.03	0.37	**0.91**	ND5	0.93	0.55	0.41	0.11	0.55	0.58	−0.64	−0.53
**0.53**	0.05	0.36	**0.87**	**0.93**	ND10	0.57	0.47	0.05	0.63	0.67	−0.66	−0.50
0.19	−0.05	0.26	**0.56**	**0.55**	**0.57**	WJAN	0.45	0.04	0.49	0.68	−0.48	−0.46
0.39	0.01	0.29	0.30	0.41	0.47	0.45	WFEB	−0.26	0.66	0.59	−0.52	−0.37
0.45	−0.05	0.17	0.24	0.11	0.05	0.04	−0.26	WSEP	−0.02	−0.05	−0.16	−0.16
**0.50**	0.00	0.35	0.42	**0.55**	**0.63**	0.49	**0.66**	−0.02	PMAM	0.79	−0.68	−0.42
0.39	−0.03	0.29	0.49	**0.58**	**0.67**	**0.68**	**0.59**	−0.05	**0.79**	POND	−0.64	−0.43
**−0.78**	0.03	−0.39	**−0.57**	**−0.64**	**−0.66**	−0.48	**−0.52**	−0.16	**−0.68**	**−0.64**	TJAN	0.90
**−0.68**	0.03	−0.33	**−0.52**	**−0.53**	**−0.50**	−0.46	−0.37	−0.16	−0.42	−0.43	**0.90**	TOCT

**Table 2:** Variables used in the logistic regression and selected statistics.

	Estimate	Std. Error	z value	P
(Intercept)	−3.70	***2.83***	−1.3	0.2
Land Cover	2.68	***0.76***	3.5	3.9E-04
ND2	4.26	***1.58***	2.7	7.1E-03
ND5	1.19	***1.50***	0.8	0.4
PMAM	−0.92	0.16	−5.6	2.1E-08
POND	4.05	0.29	13.7	<2E-16
TJAN	−0.46	0.08	−5.5	4.7E-08
TOCT	0.35	0.03	10.2	<2E-16
WJAN	−0.09	0.00	−21.9	<2E-16
WSEP	−0.80	0.11	−7.6	3.2E-14
LandCover:ND5	−1.30	***0.36***	−3.7	2.6E-04
ND2:ND5	3.73	***1.00***	3.7	2.1E-04
ND2:PMAM	−0.54	0.09	−6.1	9.1E-10
ND5:PMAM	0.31	***0.08***	3.7	2.3E-04
PMAM:POND	−0.02	0.005	−4.2	2.7E-05
LandCover:TJAN	−0.07	***0.02***	−3.2	1.2E-03
PMAM:TJAN	0.03	0.00	7.5	6.0E-14
POND:TJAN	−0.11	0.01	−13.2	<2E-16
TJAN:WJAN	0.00	0.00	22.9	<2E-16
ND2:WSEP	0.42	0.07	6.2	4.2E-10
ND5:WSEP	−0.49	0.06	−7.7	1.3E-14
POND:WSEP	0.04	0.00	10.8	<2E-16
TJAN:WSEP	0.02	0.00	8.3	<2E-16
ND2:POND	−0.54	0.13	−4.1	3.7E-05

Several of these variables change seasonally, particularly NDVI, wet days, precipitation and maximum temperature. However, the primary focus of this study is in understanding tsetse distributions in relation to climate parameters—in this case, precipitation and maximum temperature. Therefore, our results will focus largely on those variables. From Table 3, MAM precipitation is not significant alone, but instead shows significance in interaction terms with other variables. Temperature for January and October, wet days in September, and their interaction term have very strong predictive power, and indeed are highly collinear. Similarly, NDVI has a significant interaction term with land cover, and it also wields strong predictive power singly and via interactions with climate variables.

**Table 3:** Variables considered in the development of the LM.

Variable	Time span	Source	comments
Monthly maximum Temperature (TMAX)	Jan–Dec 2000	CRU TS 2.1	Kriged to 6 km
*Monthly minimum Temperature	Jan–Dec 2000	CRU TS 2.1	Kriged to 6 km
Precipitation	Jan–Dec 2000	CRU TS 2.1	Kriged to 6 km
Wet Days	Jan–Dec 2000	CRU TS 3.0	Kriged to 6 km
NDVI	2000–2007	MODIS	16-day imagery
*Cattle density	2005 estimate	FAO glbctd1t0503m	0.05° resolution
*Elevation	1996	USGS GTOPO30	
Tsetse distribution	2000	PAATIS/FAO	Continuous;modeled
Tsetse distribution	1973	PAATIS/FAO	Presence/absence
Tsetse distribution	1996	PAATIS/FAO	Presence/absence
*FAPAR (Fraction of Available Photosynthetically Active Radiation)	2000–2005	WDCC Hamburg	Redundant with NDVI
Land Cover	2000	GLC2000	Binary suitability

*- examined but ultimately omitted from the model.

Among the more robust surveys is the 1973 assessment, which we used as “ground truth.”

**Figure 2:**
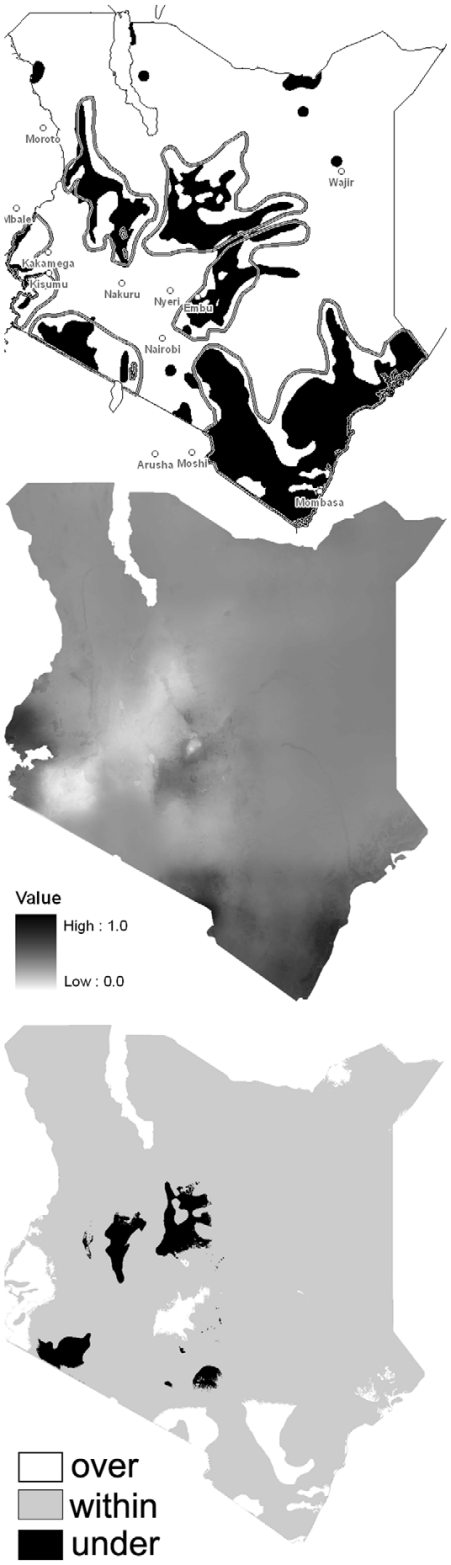
Comparison of 1973 fly abundance, modeled and observed. (A) observed, and (B) modeled tsetse presence for 1973; and (C) difference (B)-(A) showing over-, within-, and under-estimation. 1996 estimates are also outlined in grey (A) for reference.

The 1973 observations in Fig. 2 (A) are binary presence/absence, and thus the comparison with the LM probability data (B) is stark. Almost uniformly, the LM results predict a greater abundance of flies in areas where no flies (white in A) are observed, with the exception of cooler temperature regions in the highlands; for those values the LM predicts very low or zero probability of tsetse flies. The 1996 estimates, which were based on expansion of the 1973 fly belts, are also shown in Fig. 2 (A) for comparison. The high-occurrence conditions in the observed data are along the coastal areas, the slopes of Mount Kenya, the Chyulu Hills, the Mara, and in the Rift Valley. The LM captures that high occurrence well, but overestimates abundance particularly for temperatures between ∼27° and 30°C in the arid east. To compare a binary map with the LM map, we assigned LM values greater than 50% to one, and values less than 50% to zero. The resulting binary difference map in Fig. 2 (C) shows overestimation, underestimation, and a “within 50%” estimation. This comparison is admittedly coarse, but it highlights areas where tsetse distributions are poorly modeled. As the Kappa statistic is inappropriate for presence/absence comparisons, we calculated a Mapcurves GOF (“Goodness-Of-Fit”) score [25] of 0.20 for the area. “Mapcurves GOF” is a statistical tool recently developed for comparing categorical maps [25]. This statistic, used to compare the similarity of two maps, indicate that the maps “agree” for approximately 20% of the area, “agreement” being in the same 10% bin range. This level is relatively low, but the 1973 observations, which the model is compared against, have several shortcomings as well, discussed below, that makes this GOF level reasonable.

### Validating the model with FAO data from 2000

**Figure 3:**
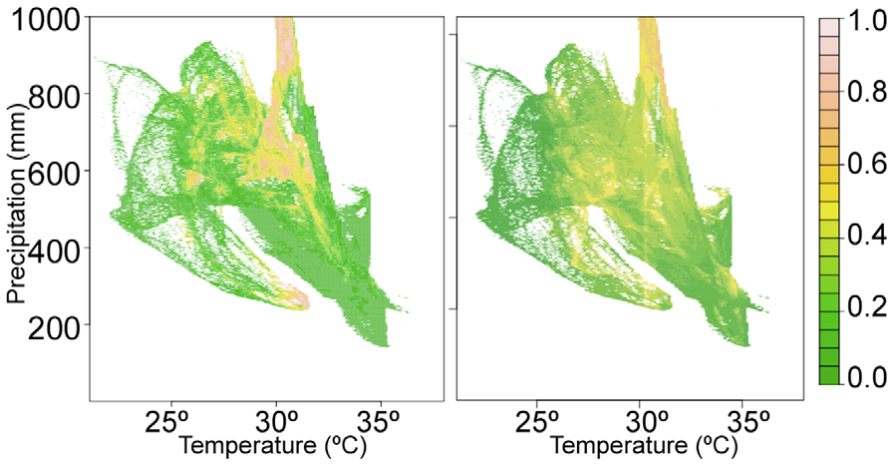
Parameter space diagrams. Year 2000 tsetse presence for observed data (left) and modeled data (right). The color scale is probability of fly presence.

Having built the model and identified areas of disagreement with FAO data, we sought to test the LM against a separate survey of tsetse data for a different time. The LM coefficients were used to construct a year 2000 tsetse distribution based on the selected variables. To evaluate the model's performance, we looked at both the parameter space and the map differences. Of particular interest is the parameter space for precipitation and maximum temperature. Fig. 3 compares the observed tsetse distribution over average annual precipitation and average annual maximum temperature parameter space showing some similarities and some distinct differences. The overall shape of the distribution is determined by the temperature and precipitation values that occurred in the kriged Climatic Research Unit (CRU) data, so both distributions have the same basic shape. The main differences lie in different color shades that represent fly abundance. For example, near the top center of both graphs are high abundance values (yellow to orange, at a probability of roughly 0.6–0.8). These high-rainfall, fly-prevalent areas correspond to the Indian Ocean coast and the modeled abundance (right panel) shows a strong correspondence with the reported fly abundance (left panel). For some precipitation and temperature combinations, the model over-predicts (e.g. for areas between 33°–35°C) or underpredicts (for areas below ∼26°C). The underpredicting areas are primarily in savanna regions southeast of Nairobi and in southwestern Kenya. The areas overpredicted by the model lie near the Ethiopian border and in isolated pockets north of Nakuru.

**Figure 4:**
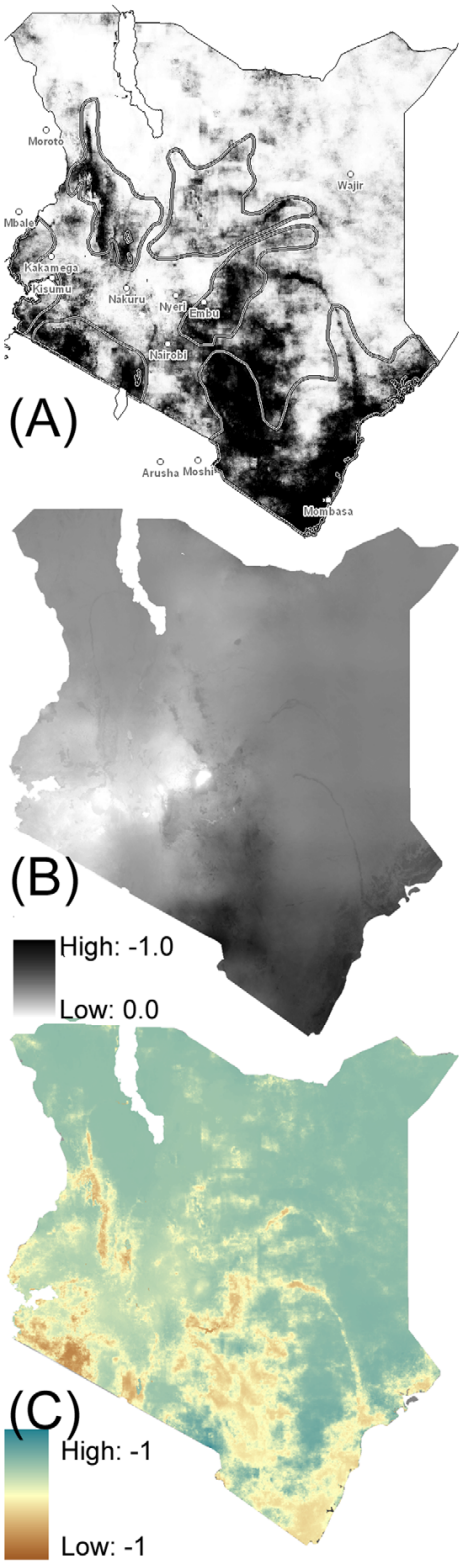
Comparison of 2000 fly abundance, modeled and observed. (A) Observed and (B) modeled tsetse presence for 2000. (1996 estimates are also outlined in grey for (A) for reference); (C) difference (B-A).

FAO and LM estimates, each shown in Fig. 4 (A and B) along with the difference between the two (C), contrast markedly in geographically distinct areas. Generally, the FAO estimates in (A) adhere to a more binary distribution—almost presence-absence— whereas the LM estimates have more mid-range values. The differences (Fig. 4(C)) point to areas of significant departure between the two estimates, with LM values underestimating PAATIS (the Programme Against African Trypanosomiasis Information System) (brown areas) along major waterways, national park areas, and the well-documented fly belt along the Athi River Valley. The LM model does estimate some fly population in these areas; however, the estimates are muted compared to the FAO data. In some cases (e.g. Kenya's coast near the Somali border) the climate circumstances are suitable for tsetse survival but FAO estimates show low to no abundance. Agreement between the two estimates (white areas) is best in the highland areas, the Rift Valley, Tsavo and the near-coastal areas. A Mapcurves GOF score [25], a measure of map similarity, was calculated to be 0.22, which is similar to the Mapcurves GOF score for the 1973 data. We also calculated a 0.14 Kappa statistic.

### Projection under enhanced GHG

**Figure 5:**
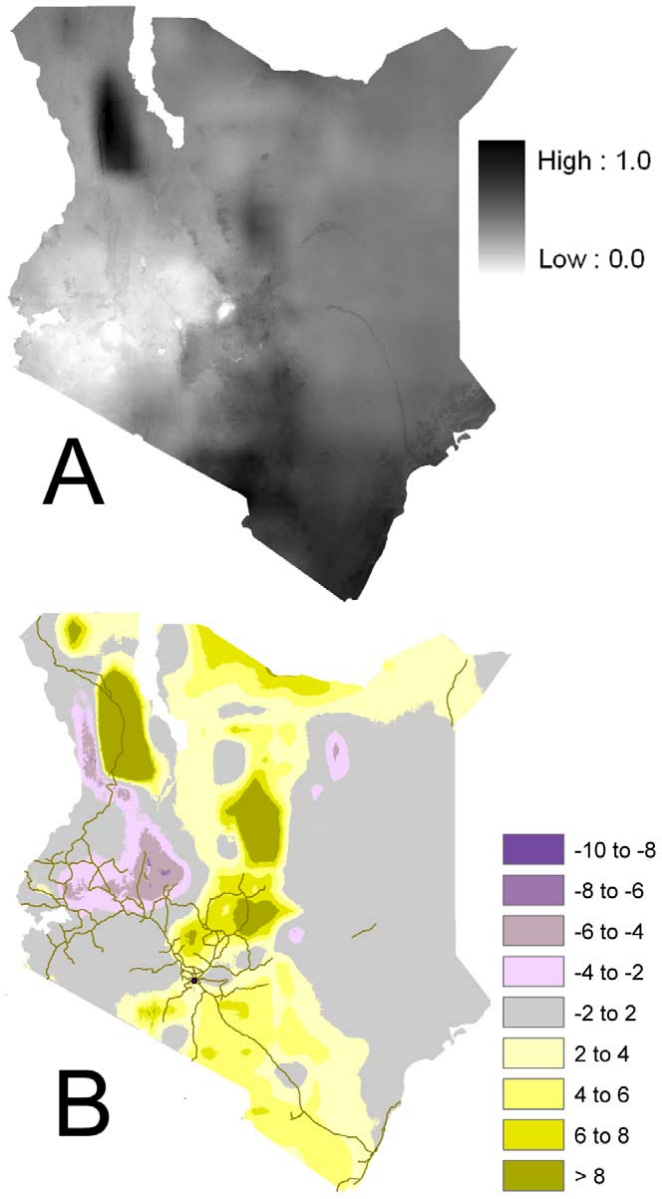
Changes in tsetse abundance due to greenhouse gases. (A) Projected tsetse presence under 2050 greenhouse gas levels, and (B) percentage change in tsetse presence for elevated greenhouse gases.

We developed a simple estimate of potential tsetse abundance under a single case of elevated GHG based on the model. We applied climate perturbations (future minus current conditions) to the Climatic Research Unit at the University of East Anglia (CRU) 2000 data, which resulted in generally warmer and wetter conditions. The model response was dramatic; as can be seen in Fig. 5 (A), the model indicates suitable habitat for tsetse would be found throughout the heavily populated higher elevations of Kenya.

The map in Fig. 5 (B) shows the difference in projected minus current abundance (Fig. 5 (A) – Fig. 4 (B)) to show general areas of increased and decreased fly abundance. In addition, increased rainfall in the Lake Turkana region shifts the precipitation/temperature regime towards much higher abundance values in the diagram in Fig. 3. The anomalous rise west of Lake Turkana is likely a consequence of drastic rainfall increases in the region, which may be due to errors in climate projection. A similar regime shift occurs along the higher elevation isocline of the eastern highlands, ranging from Mombasa and Kilimanjaro towards Tsavo National Park (yellow areas), and in fly belts north and east of Mt. Kenya up to Marsabit (dark yellow). This shift is towards much higher temperatures at higher elevations (to the upper right of Fig. 3, right panel) where fly abundance is currently low. The Kisumu area on to the Rift Valley and north along the Uganda border show decreases in tsetse abundance likely due to warmer temperatures and lower rainfall. Since this is a single projection into the future, however, the overall trends are more salient than the specific locations. In Fig. 5 (B), the model shows tsetse changes following a clear topographic gradient along the eastern scarp stretching from Marsabit to Mt. Kilimanjaro. A decrease in tsetse (increase) in abundance at marginally higher elevations suggests an “uphill migration” along most topographic gradients—including along the Ethiopian border—with only a few areas near the Indian Ocean coast exhibiting a non-topographically-driven shift in tsetse abundance.

## Discussion

### Aspects of building and validating the model

This logistic regression model was used predict tsetse abundance based on climate and biophysical characteristics. The coefficients (in Table 2) show some strong positive relationships between tsetse abundance and land cover, February NDVI and “short rains” precipitation. During February, at the end of the dry season, fly abundance should be relatively low in dry areas. Thus, a strong relationship is expected between tsetse abundance and the remaining wet areas represented by these three variables. Negative relationships exist between tsetse abundance and dry season wet days (WJAN and WSEP); this follows from sparse tsetse abundance in areas that receive dry season precipitation, which tend to be dominated by agriculture or high-elevation cool climates. Some coefficients in Table 2 show negative relationships with tsetse abundance that are unexpected, particularly “long rains” precipitation. However, “long rains” precipitation is also highly correlated with agriculture, and agricultural areas are negatively correlated with tsetse abundance as described earlier. Some of the independent variables' coefficients are easy to explain, while others, particularly interaction terms, are less transparent to interpretation.

This type of model is merely predictive, and the coefficients determined by the LM may or may not reflect actual relationships between variables and tsetse abundance. As a result, some regions in the LM results may have errors that can be attributed to a specific cause while other may not. For this reason, we have not attempted to explain or evaluate the roles of specific variables for influencing specific high or low values of tsetse abundance. However, the general trends in tsetse abundance are broadly explained by actual relationships between variables that are known. In some cases— like “long rains” precipitation (PMAM) being a negative predictor, for example— a variable can “stand in” for agriculture or another unknown characteristic. Our selection of model was done by repeatedly examining lots of models to rule out (where possible) clearly unrealistic or illogical coefficients, e.g. a negative coefficient for suitable land cover. Thus, the independent variable coefficients of the final model appear to reflect real relationships with tsetse abundance. This study then tested the resulting tsetse abundance maps against other estimates.

We compared our LM results with the FAO/PAATIS model, which has not been validated. Comparing our LM model to the FAO data assumes that the FAO data correctly capture all instances of fly presence. Thus, our comparisons may be artificially low or high in some regions. Since the main objective of this study is to produce a new tsetse abundance map, differences should be expected. Indeed, by understanding areas of disagreement, we can infer potential areas for on-the-ground fly presence testing that would distinguish between the utility of each model for a given area. In this case, we find some differences and potentially new tsetse distributions.

Mapcurves GOF [25] scores indicate that the FAO estimates and the LM estimates have some measure of agreement. These similarities arise from both models being driven by similar datasets and from both being constructed in similar ways. However, important differences exist, and these differences may point the way towards building an explanatory model. For example, both models use vegetation (NDVI) as a predictor variable, but the FAO model also uses infrared reflectance and uses a Fourier approach, among other differences. Ecological areas in the FAO model were represented by these remotely sensed data, whereas in the LM we tested actual land cover as a predictor variable. Including land cover in future models is sound because tsetse flies are rarely found in certain types of land cover, e.g. crop-growing areas where preferred food sources are not available. The FAO model's stated goal was “to produce continent wide predictions of the probability of presence of twenty three tsetse species”, whereas our goal differed in region and in species specificity. The maps have relatively low GOF scores, but low GOF scores may merely indicate these different goals.

The disagreement between models means that significant variability exists in even simple models driven by fairly similar datasets. Any modeling at the national scale will need validation data. Our efforts to validate the model against 1973 data yielded a similar GOF score (0.20). The similarity in the GOF scores for FAO, 1973 and 2000 data points to overlaps among all three estimates; we interpret this (along with comparing the map similarities) as meaning that the LM captures major fly abundance areas well, but not necessarily lower abundance areas. There are specific areas in Fig. 5 that the LM captures poorly—mainly river valleys and cool, wet areas—where lack of a suitable predictor variable like soil moisture in the LM led to the model's failure to develop any predictive power for a specific type of habitat, like river microclimates in otherwise arid regions. Using a different data set than the FAO would probably produce a different set of estimated coefficients, but given the dearth of available data it is unlikely that the Mapcurves GOF scores would improve greatly. Year-to-year variation in tsetse populations, food source distributions, and other sources of variability make a purely logistic model limited to identifying suitable places for tsetse abundance, but not necessarily for tsetse presence. In particular, an averaged 8-year NDVI seasonality (required for use with 1973, prior to remote sensing) clearly decreases signal-to-noise ratio and is a significant source of error. The best application of the LM estimates is to aid in locating potentially suitable areas of tsetse habitation. This information, in conjunction with estimates of seasonal tsetse refugia, can help focus control efforts. These areas are identified as higher-elevation zones adjacent to current fly belts, along with regions that may experience wetter-than-normal conditions in the future. Repeated fly surveillance in these areas would aid both in model improvement and in possible early detection of fly belt expansion.

The lack of numerous tsetse datasets for modeling is a severe constraint. Currently, insufficient fly-trap data exist to validate either model except perhaps in isolated areas. Significant differences are evident between the FAO data and the 1996 “fly belt” maps. Both the FAO probabilities and our LM probabilities are not validated against objective data, and the 1996 estimates, though approximate, are perhaps the closest thing to objective data. Some areas may have high tsetse fly abundance, but data are lacking in the 1996 fly belt map (e.g. Kenya's coast near the Somali border) even though the climate circumstances are suitable and anecdotal reports supportive. Implementing trapping efforts is warranted in locations suggested by these model results and other sources.

### Potential new tsetse sites

Potential new areas of tsetse abundance suggested by the LM point to elevation/climate gradients. These gradients may be useful for helping establish monitoring transects, particularly in areas that are currently thought to be tsetse-free. Although little early detection of climate effects can be directly attributed to GHG in Kenya [26], real-time detection of tsetse habitat shifts due to weather and climate factors would be identifiable by GIS-based models and carefully controlling for other factors that could enhance changing habitat, like land use and changing food source distributions (i.e. cattle and wildlife). We do not recommend using Fig. 5 as a projection of future tsetse populations because of three considerations: 1) it is derived from climate impacts of a single GCM, 2) it does not contain any projection of future land use or vegetation distribution, and 3) it lacks explanatory power. However, the model does identify likely gradients for monitoring fly presence/absence; with deterministic vector-based modeling as well as additional climate perturbations from different GCMs, this LM approach may aid in more statistically robust predictions of tsetse abundance. Our next steps will include modeling improvements and interpreting the drivers behind specific changes in tsetse abundance. This leads us to call for more frequent data collection of time-varying climate and vegetation conditions that affect tsetse abundance. In addition, we recommend increasing funding of fly surveillance efforts to assist managing tsetse populations more effectively.

## Materials and Methods

### Logistic Regression Model

Our approach to modeling *Glossina spp.* distributions began with determining which variables would be best to use in the regression model. To avoid needless collinearity created by simply using all the variables available, we followed recommendations from Burnham and Anderson [27], which include omitting unrealistic relationships, positing a small set of models first, and avoiding a “just the numbers (let the computer figure it out)” approach. First we examined simple one-variable models to construct a set of meaningful variables for predicting tsetse abundance. Many of these variables were similar to the FAO model (see below). For rainfall, many months had highly similar patterns driven by the “long rains” and the “short rains”. For this reason, precipitation was aggregated together for March through May (PMAM) and for October through December (POND). All monthly temperatures were heavily correlated with one another. Since temperature was also highly correlated with elevation, and because we want to construct a map of tsetse abundance based on climate parameters, we omitted elevation and included temperatures. We retained months where fly mortality is typically highest: January and October, immediately prior to rainy season onset. As a representative of dryness, we included the CRU wet days index for the driest months: January, February, and September. Fraction of Available Photosynthetically Active Radiation (FAPAR) was strongly correlated with NDVI, and was thus omitted at the outset (thus not included in Table 1 or Table 2) since minimum temperature and maximum temperature were also very tightly correlated. Similarly, to avoid multicollinearity as much as possible, we only used maximum temperature to represent temperature since it is a more common driver of temperature-related tsetse mortality in Kenya than cold temperatures, which occurs only in the highlands.

NDVI data allowed for seasonal changes in land cover to be incorporated into the model. However, since NDVI data for 1973 are unavailable, it would be inappropriate to use 2000 NDVI data alone. To resolve this, we constructed annual average NDVI data from 2000–2007 to represent general seasonal patterns. This averaged NDVI was used for 1973, 2000, and future modeled tsetse distributions. This necessarily reduces model accuracy but allows for inclusion of seasonal greening patterns. Elevation/Topography data (ultimately not used) were from the USGS GTOPO30 digital elevation model (DEM). The FAO 2005 livestock census of cattle was ultimately not used. The 2000 FAO fly data are based on a model, and we are thus using a logistic model to predict another model; however, the 2000 data are the best available, leaving little alternative. Unfortunately, no monthly tsetse fly data were available for any year. Tsetse data for 2000 were gathered and/or estimated by PAATIS [28] as part of work for the FAO and IAEA. 2000 FAO data overlap with the remote sensing era. To compare the maps we used the Mapcurves GOF score [25] by sorting data from both maps into 10%-increment bins for the GOF procedure.

Variable names are as follows:

*ELEV = elevation (m)

LC = land cover suitability (binary; 1 = suitable, 0 = not suitable)

*COW = cattle density (head/km^2^)

ND2 = Normalized Differential Vegetation Index for February (unitless)

ND5 = Normalized Differential Vegetation Index for May (unitless)

*ND10 = Normalized Differential Vegetation Index for October (unitless)

*WJAN = CRU wet days index for January (days)

WFEB = CRU wet days index for February (days)

WSEP = CRU wet days index for September (days)

PMAM = sum of precipitation for March, April, and May

POND = sum of precipitation for October, November, and December

TJAN = average monthly temperature for January

TOCT = average monthly temperature for October

*ultimately removed from the AIC process and the final model

Following the principle of parsimony, we constructed a correlation matrix to remove highly correlated variables. The correlation matrix, shown in Table 1, was used to diagnose this multicollinearity; here we omitted elevation, October NDVI, and wet days in January. Correlations greater than or equal to 0.5 are in bold. Next we applied a stepwise algorithm called “stepAIC” in R to aid in model selection. This procedure iteratively adds and/or subtracts individual variables or combinations of variables to seek the lowest AIC score. Lower AIC scores are judged the most appropriate models. We only considered the 9 first order terms and several second-order interaction terms, for a total of 35 terms. Stepwise AIC was applied to this initial model.

The following 24 term final model selected through AIC [27] was:
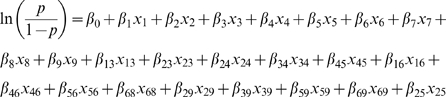
where the x_1_ = LC, x_2_ = ND2, x_3_ = ND5, x_4_ = PMAM, x_5_ = POND, x_6_ = TJAN, x_7_ = TOCT, x_8_ = WJAN and x_9_ = WSEP.

And the coefficients ß_i_ are given in Table 2. The dependent variable *p* is tsetse abundance on a scale from zero to one. This exploratory approach mainly focuses on developing a new approach to modeling the tsetse spatial distribution and does not seek to explain causality, nor predict or diagnose tsetse population. The resulting coefficients of the final model determined by the stepwise AIC process are given in Table 2. Descriptions of the variables considered are outlined in Table 3.

Following the basic structure of Wint and Rogers [22] and Wint [23], this study used logistic regression modeling rather than discriminant analytical/maximum likelihood analysis [28], [29]. We depart from the Wint and Rogers [22] approach by omitting highly correlated variables and removing illogical relationships. We used the R software and several different potentially useful datasets were gathered. All data were initially projected to geographic coordinates. CRU data (TS 3 [30]) originally at 0.5° resolution, were kriged to 6km following Goovaerts [31], [32], thus 213930 data points were utilized from the original 1283580 data points at 1 km resolution.

Our study considered three time periods and for these, we:

1. Developed a regression model based on 1973 climate data;
2. Assessed the validity of the model based on current (2000) data.
3. Estimated tsetse abundance based on projected climate data under elevated GHG.

To project distributions of tsetse under elevated GHG conditions, we extracted climate data from the National Center for Atmospheric Research's Community Climate System Model (CCSM) version 3.0, namely, precipitation and temperature data for the decades 2000–2009 and 2050–2059. These data were used to drive the Regional Climate Modeling System (RAMS, version 4.4) [33] at 36 km grid spacing and 32 terrain-following vertical levels at a 60-second time step. RAMS phenology was constructed from remotely sensed NDVI, and land cover from the Global Land Cover 2000 dataset. More details on the climate model configuration can be found in Moore et al. [34]. These decades were selected to evaluate effects of elevated GHG on climate variables versus those at current GHG levels. To determine the possible extent of tsetse expansion, we used data from the Intergovernmental Panel on Climate Change A1B scenario [26]. A1B makes no adjustments from “Business-As-Usual” until CO_2_ concentrations reach 720 ppmv (very aggressive), and as such provides something like an upper bound to potential tsetse distribution. These data from the climate models were **not** directly used in the LM as inputs; rather, the difference (elevated minus current) was treated as a perturbation added to the CRU data for 2000. Since currently, climate models exhibit no skill at decadal timescales, it is impossible to assert that these data can give rise to projections of tsetse distribution. Instead, we treat these results as a testing of a new modeling approach; and, as identifying areas of sensitivity to tsetse encroachment instead of actual projections of future tsetse distributions. Indeed, without accurate sampling data, model artifacts can arise and severely limit the useful application of model results by agencies and livestock holders [35]. Anticipating future tsetse distributions may allow for targeted control efforts.
